# Radiographic analysis of adult ankle fractures using combined Danis-Weber and Lauge-Hansen classification systems

**DOI:** 10.1038/s41598-020-64479-2

**Published:** 2020-05-06

**Authors:** Shu-Man Han, Tian-Hao Wu, Jin-Xu Wen, Yong Wang, Lei Cao, Wen-Juan Wu, Bu-Lang Gao

**Affiliations:** 1 Department of Radiology, The Third Hospital of Hebei Medical University, Shijiazhuang, China; 2 Department of Radiology, Gucheng County Hospital of Hebei Province, Gucheng, Hebei China

**Keywords:** Trauma, Bone imaging

## Abstract

This study was to analyze ankle fractures for determining the epidemiology, types, distribution, possible mechanisms and diagnosis precision. Between January 2013 and December 2017, all Chinese patients older than 16 years of age with ankle fractures excluding old ankle fractures and pathological fractures in a tertiary care hospital were analyzed by using the Danis-Weber and Lauge-Hansen classification systems. Among 3952 patients with ankle fractures, 1225 fractures (31%) were Danis-Weber type A, 1640 (42%) were type B, 751 (19%) were type C, and 336 (9%) were perpendicular compression fracture. There were 1949 fractures on the left side and 2003 on the right with no significant difference (P > 0.05). Male patients between 16 and 50 years of age and women over 50 years had a higher incidence of ankle fractures accounting for 38.4% (1517/3952) and 22.2% (800/3952), respectively. Posterior malleolar fractures, fibular fractures above the inferior tibiofibular joint and Tillaux fractures were easily missed in the diagnosis, with 38 fractures (0.96%) being missed in the diagnosis. In conclusion, young and middle-aged men and older women have a higher incidence of ankle fractures, and use of the Lauge-Hansen and Danis-Weber classification systems can better help assessing the varied and complex ankle fractures, predicting the injuries, increasing diagnostic precision and decreasing misdiagnosis rate.

## Introduction

The unique pattern of anatomy of the ankle and the functional relationship with the foot make the ankle highly susceptible to injuries^1–5^. Ankle fracture is a common injury with recent studies demonstrating an annual incidence of 120–150 fractures per 100,000 persons^6^. Ankle fracture is one of the commonest injuries treated by orthopedic surgeons, accounting for 9% of all fractures and 36% of all lower extremity fractures in the United States, and the rate is still increasing particular among elder citizens because of ageing-associated increases in fragility fractures^7,8^. Treatment of the ankle fracture depends on careful recognition of the range of bone injuries and damage to the soft tissue and ligaments. Evaluation of ankle fractures mandates detailed history of disease, physical examination, proper radiographic examination and initial treatment choices, and successful treatment lies in correct diagnosis of this fracture followed by anatomical restoration of the bone structures involved for tibiotalar joint reconstruction.

There are several types of classification for ankle fractures. Ankle fractures were firstly classified as unimalleolar, bimalleolar or trimalleolar^5,9^. However, this classification does not distinguish unstable and stable injuries even though it is intuitive and easy to reproduce. Through cadaver experiments, Lauge-Hansen suggested a classification system which correlates the lines of ankle fractures with specific traumatic mechanisms^10^, with the fractures being classified into four groups: supination-adduction, supination-external rotation, pronation-external rotation, and pronation-abduction. The first term specifies the position of foot at injury and the second indicates the direction of the force applied to the foot at injury^5^. The Lauge-Hansen classification system indicates that the most frequent type is the supination-external rotation pattern with a prevalence of 40%-75% of all emergency ankle fractures. This classification can be used to accurately diagnose the range of injury, identify associated ligament injuries and determine the severity of the injury as well as the degree of instability. However, it also has one disadvantage of complexity for routine use in daily life in the hospital^11^. Combination of the Lauge-Hansen classification system with the Danis-Weber system would make clinical application easier. The Danis-Weber classification system was proposed by Danis and Weber according to the location of the primary fibular fracture line, with the fractures being divided into three groups: type A (below the syndesmosis level), type B (at the syndesmosis) and type C (above the syndesmosis)^5^. This classification system is simple and easy of reproduction, however, it does not consistently predict the scope of injury in the tibiofibular syndesmosis since types B and C can be managed in a similar approach regardless of location of fracture. This classification does not consider the state of the structures on the medial side, either, and it is impossible to compare prognosis or evolution^12,13^. We hypothesized that the combination of the Danis-Weber and Lauge-Hansen classification systems could be used to investigate the patterns of ankle fractures and patterns which might be easily misdiagnosed and to guide clinical treatment. This study was consequently performed to investigate the role of the combination of the Danis-Weber and Lauge-Hansen classification systems in diagnosing ankle fractures.

## Patients and methods

This retrospective study was approved by the ethics committee of the Third Hospital of Hebei Medical University with all the patients or their legal guardians given their signed informed consent for imaging and management. All methods were performed in accordance with the relevant guidelines and regulations. Between January 2013 and December 2017, all Chinese patients with ankle fractures in a tertiary care hospital were enrolled with the inclusion criteria being older than 16 years of age and ankle fractures confirmed by radiographic examination. Exclusion criteria were patients with old ankle fractures and pathological fractures. Two senior radiologists independently evaluated the clinical and imaging data of ankle fractures including age, sex, location and types of fracture, and space of joint. When in disagreement, a third radiologist would be involved to reach an agreement. The patients were divided into eight age groups, with 16–20 years of age as one age group, ≥81 as an age group, and every ten years as an age group between 21 and 80 years.

**Figure 1:**
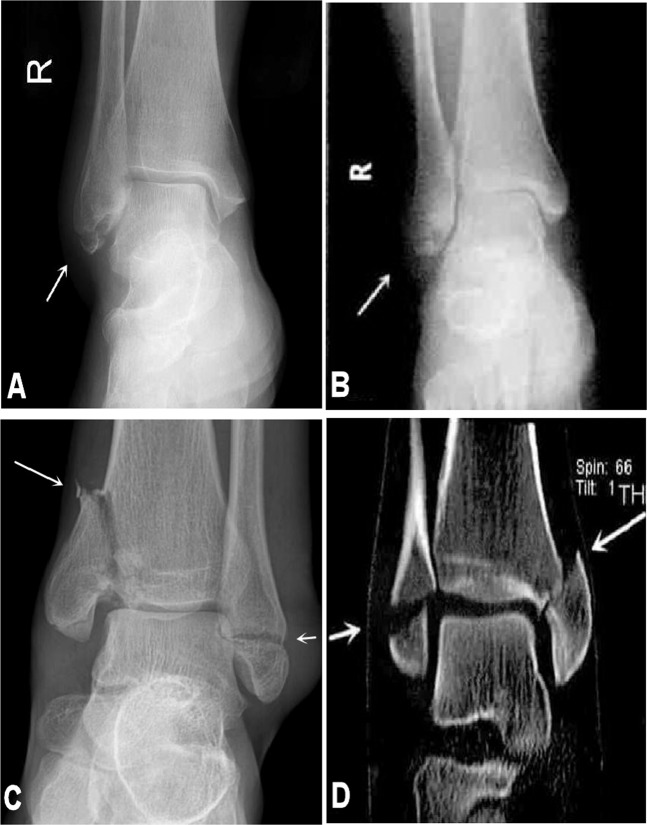
Lateral and medial malleolus fractures. (**A**,**B**) The lateral malleolus fracture was shown (arrow) with the fracture line below the inferior tibiofibular joint level. This is Weber type A stage 1 and supination-adduction stage 1 of the Lauge-Hansen classification. (**C**,**D**) The lateral malleolus fracture (short arrow) and the medial malleolus fracture (long arrow) were demonstrated. According to the Danis-Weber classification, this is Weber A stage 2. According to the Lauge-Hansen classification system, this is supination-adduction stage 2. The lateral malleolus fracture line is transverse below the inferior tibiofibular joint level. The medial malleolus fracture is higher than the lateral malleolus fracture with the medial malleolus fracture line being vertical or oblique.

**Figure 2:**
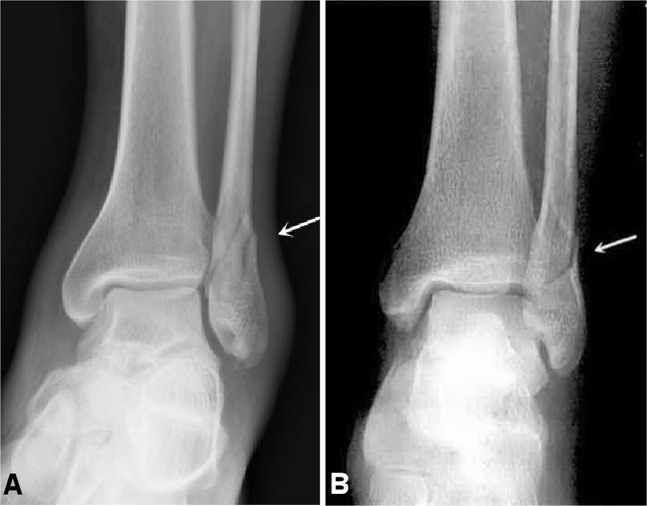
Fibular fracture (**A**,**B**) is shown at the inferior tibiofibular joint level (arrow). It is stage 2 of Weber Type B fracture or of supination-external rotation of the Lauge-Hansen classification.

**Figure 3:**
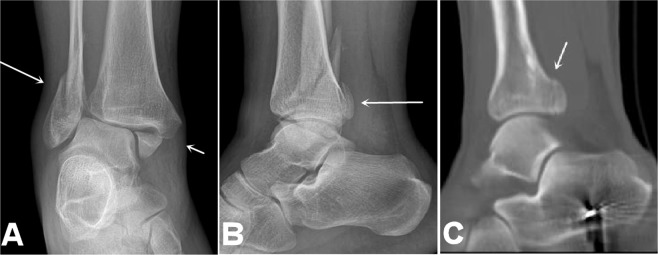
Lateral and medial malleolus fractures. (**A**) Anteroposterior radiograph shows lateral and medial malleolus fractures with inhomogeneous density at the distal tibial end which suggests posterior malleolus fracture. The inferior tibiofibular joint space is increased. B&C. Lateral radiograph (**B**) reveals the posterior malleolus fracture (**B**) which is confirmed by computed tomographic scan (**C**). This is stage 4 of the Weber type B fracture or of the supination-external rotation of the Lauge-Hansen classification.

**Figure 4:**
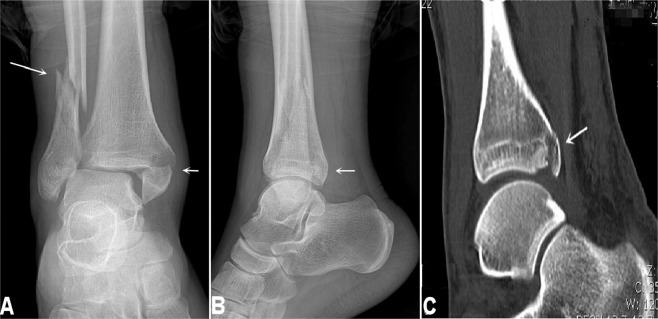
Medial malleolus fracture and high-location fibular fracture. (**A**). The medial malleolus fracture (short arrow) and the fibular fracture (long arrow) are shown. (**B**). Lateral radiograph shows inhomogeneous density at the posterior malleolus, suggesting possible fracture at this site. (**C**). Computed tomographic scan confirms the posterior malleolus fracture (arrow). This is stage 4 of the pronation-external rotation type of the Lauge-Hansen classification. The medial malleolus fracture line is transverse. The fibular fracture is oblique or spiral and is frequently located above the inferior tibiofibular joint level.

**Figure 5:**
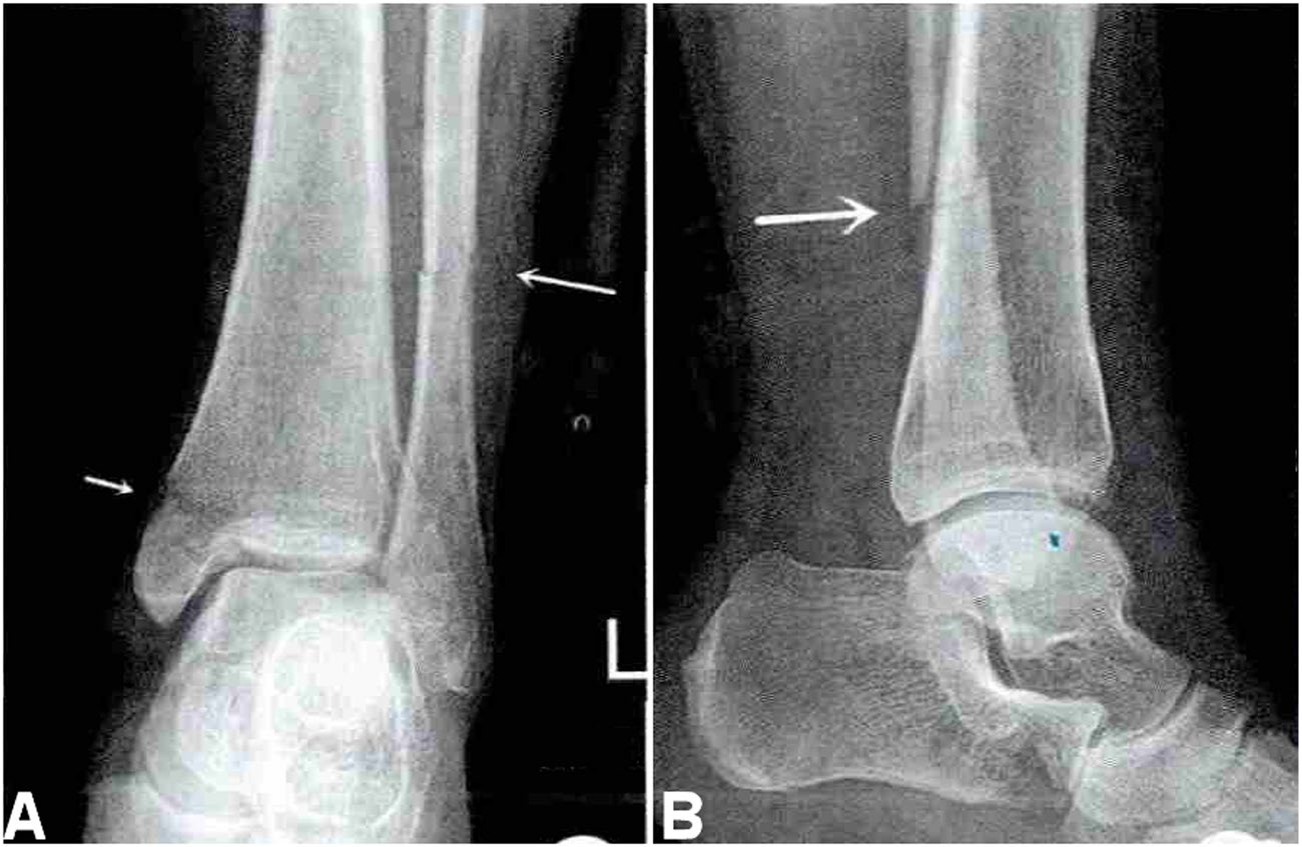
Medial malleolus fracture and fibular fracture above the lower tibiofibular syndesmosis. The medial malleolus fracture (short arrow in **A**) and the fibular fracture (long arrow in both **A**,**B**) are demonstrated. This is stage 3 of the Lauge-Hansen pronation-abduction fracture. The fibular fracture line is transverse above the inferior tibiofibular joint level sometimes with small bone fragments but without accompanying posterior malleolus fracture.

Firstly, the ankle fracture was categorized using the Danis-Weber classification system, and then, the fractures were further evaluated with the Lauge-Hansen classification. Danis-Weber type A corresponded to the supination-adduction type in the Lauge-Hansen system with the fracture line below the inferior tibiofibular joint level (Fig. 1). Danis-Weber type B corresponded to the Lauge-Hansen supination-external rotation or partial pronation-abduction type with the fibular fracture line at the inferior tibiofibular joint level (Figs. 2, 3). Danis-Weber type C corresponded to the Lauge-Hansen pronation-external rotation and partial pronation-abduction type with the fibular fracture line above the inferior tibiofibular joint level (Figs. 4, 5). Perpendicular compression fracture type was compression fracture of the ankle.

### Statistical analysis

Statistical analysis was performed with the SPSS software (version 24, IBM, Chicago, IL, USA). Descriptive statistics (percent, mean and standard deviation, and median) were used and tested with the ANOVA test. The significant P value was set at P < 0.05.

## Results

**Figure 6:**
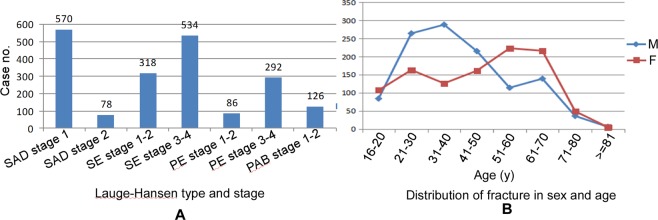
Distribution of ankle factures in the Lauge-Hansen type and stage, sex and age. SAD, supination-adduction; SE, supination-external rotation; PE, pronation-external rotation; PAB, pronation-abduction.

A total of 3952 patients with ankle fractures were enrolled including males and females with an age range of 16–94 years (mean 42.3). The composition of Danis-Weber fracture types A, B and C and perpendicular compression fracture was 31% (1225/3952), 42% (1640/3952), 19% (751/3952) and 9% (336/3952), respectively (Fig. 6). There were 1949 fractures on the left side and 2003 on the right side with no significant difference (P > 0.05).

Among 1225 patients with Danis-Weber type A (Table 1, Figs. 1 and 6A), 1065 cases belonged to the Lauge-Hansen stage 1 accounting for 87% in this type while 160 cases were in stage 2 (13.0%). Predominance was found in female patients with a male-to-female ratio of 0.64:1 (478/747). Forty seven patients had further computed tomography (CT) scanning accounting for 3.8% because of no definitive diagnosis or suspect of ankle fracture or for treatment planning. Twelve patients had ankle dislocation or subluxation (0.98%).

**Table 1:** Data for Lauge-Hansen groups and stages.

Variable	supination-adduction		supination-external rotation		pronation-external rotation		pronation-abduction	Total
Stage	1	2	1–2	3–4	1–2	3–4	3	
No.	1065	160	491	820	147	488	445	3616

Note: The number of 3616 indicates patients whose fractures belong to the Lauge-Hansen groups and stages. There are still 336 patients who had perpendicular compression fractures which did not fall into the Lauge-Hansen categorization.

Among 1640 patients with Danis-Weber type B fractures (Figs. 2, 3), 491 cases belonged to the Lauge-Hansen stages 1 and 2, accounting for 30%, and 820 cases (50%) to stages 3 and 4, with no significant difference in sex prevalence (male/female: 781/859 or 0.943:1, P > 0.05). CT scanning was performed in 627 cases (38%), and 698 cases (43%) had luxation or subluxation. There were 1311 cases (80%) in the Lauge-Hansen stages 1–4 and 329 (20%) cases in the stage 3 of pronation-abduction. The stages 1–2 of the pronation-abduction were difficult to be distinguished from those of the pronation-external rotation and were subsequently designated to Danis-Weber type C for analysis.

**Figure 7:**
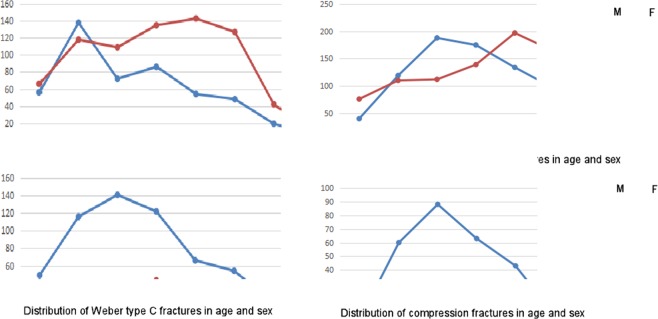
Distribution of ankle fractures in Danis-Weber classification and of compression fractures in age and sex. There were 1225 ankle fractures in Danis-Weber type A, 1640 in type B, 751 in type C, and 336 in compression fractures.

Among 751 patients with Danis-Weber type C fractures (Figs. 4, 5), 147 patients (19.6%) belonged to stages 1–2 of both Lauge-Hansen pronation-external rotation and pronation-abduction groups because of difficulty in distinguishing different groups, and 604 patients (80%) belonged to stages 3–4 including 488 cases in stages 3–4 of the pronation-external rotation and 116 in stage 3 of the pronation-abduction group. This type had a predominance in males with a male-to-female ratio of 3.1:1 (568/183). CT scanning was performed in 411 cases (55%), and luxation or subluxation occurred in 384 cases (51.1%). The male to female ratio was 4.17:1 (271/65) in 336 patients with perpendicular compression fracture. The distribution of the ankle fractures in the Danis-Weber classification and of the compression fractures was shown in Fig. 7.

Analysis of patients at different ages demonstrated 1517 male patients between 16 and 50 years of age, accounting for 38% of all cases, and 800 female patients over 50 years, accounting for 22% (Table 2 and Fig. 6B). Among 3952 cases with ankle fractures, 35 cases (0.89%) with posterior malleolus fractures were missed at the initial plain radiography but confirmed by CT scanning including 13 cases with occult posterior malleolus fractures which could not be demonstrated by plain radiography. Six patients (0.15%) with a relatively higher location of fibular fractures were not found in the initial ankle joint radiography but were discovered in repeated plain radiography covering the middle and superior segments of fibula three days to half a month later. Twenty one patients (0.53%) with Tillaux fractures confirmed by CT scanning were not found in initial plain radiography including 11 patients with occult Tillaux fractures which could not be displayed by plain radiography. In statistics, missed diagnosis was defined as those fractures which could be found in both plain radiography and CT scanning but was not reported in the initial diagnosis, and the total missed diagnosis rate was 1% (38/3952) in the posterior malleolus fractures, fibular fractures at a relatively higher location and Tillaux fractures. Those occult fractures which could not be recognized on plain radiographs were not included in the rate of missed diagnosis.

**Table 2:** Sex and age distribution patients with ankle fractures.

Sex	16–20 y	21–30 y	31–40 y	41–50 y	51–60 y	61–70 y	71–80 y	≥81 y	Total
Male	148	434	489	446	297	205	67	12	2098
Female	173	269	276	336	371	309	106	14	1854

Note: The total number is 3952 with ankle fractures.

## Discussion

### Major findings

In this study, we analyzed a large number of patients with ankle fractures and found that Danis-Weber type B is the most common ankle fracture followed by Danis-Weber type A, type C and perpendicular compression fracture, with a similar incidence on the left and right side. Ankle fractures occur most frequently in the young and middle-aged men between 21 and 50 years (peaked at 30–40 years) and old women between 50 and 70 years of age. Combination of the Danis-Weber and the Lauge-Hansen classification systems can help decreasing the misdiagnosis rate and increasing the correct diagnosis rate in clinical evaluation of ankle fractures.

### Mechanism and types of ankle fractures

It is extremely important to know the precise mechanism of ankle fracture for accurate evaluation of the fracture form. The Lauge-Hansen classification system depicts firstly the foot position at the time of injury and, secondly, the external deforming force on the ankle^14^. When the ankle is in one of the following locations at injury: supination (inversion) and pronation (eversion), three deforming forces may be exerted: adduction, abduction and external rotation to cause fractures with corresponding mechanisms of injury: pronation-abduction, pronation-external rotation, supination-adduction, and supination-external rotation. In both injury mechanisms of pronation-external rotation and supination-external rotation, a posterior malleolar fracture may take place. In stage 4 of the pronation-external rotation injury mechanism, posterior malleolar and fibular fractures may happen together with or without medial malleolar fractures. On the other hand, posterior malleolar and fibular fractures may take place with no medial malleolar fractures in stage 3 of the supination-external rotation injury mechanism, or a trimalleolar fracture may occur in stage 4 of the supination-external rotation injury mechanism^15^.

In our study, the commonest type of ankle fracture was Danis-Weber type B accounting for 42% of all the fractures. Moreover, 50% (820 cases) belonged to stages 3 and 4 of the Lauge-Hansen classification with 43% of luxation or subluxation. This indicates a greater deforming force leading to this type of fracture. Danis-Weber type A was the second commonest ankle fracture with most patients (87%) in Lauge-Hansen stage 1 and a dominance in females, which suggests a deforming force relatively weaker than that causing Type B fracture. In this type of fracture, some patients may tolerate the trauma and will not seek for treatment, resulting in a lower incidence of Type A fracture. In stage 2 of Type A fracture, the medial malleolus fracture was usually caused by pushing force from the huckle-bone with the fracture line usually locating higher than that for Type B and C fractures and with complete triangular ligament. In Danis-Weber Type A and B ankle fractures, the foot was in the supination position in 72.5% of ankle fractures. When the fracture occurred in this position, the foot was in plantar flexion with the huckle-bone moving forward, and because the lateral malleolar ligament and bone structures were not as strong as on the medial side, these two types of ankle fracture subsequently took place. In Danis-Weber type C fracture, the foot was in the pronation position, and a strong pronation force of the body will cause this type of fracture^15,16^.

### Prevalence of ankle fractures

This study found that the young and middle-aged men between 21 and 50 years and old women between 50 and 70 years have a greater incidence of ankle fractures. Young and middle-aged men are the major workers engaging in heavy traffic and construction work in China, and they are exposed to the injury factors more frequently than people in the other age groups. Furthermore, these men tend to participate in sports more frequently than people in the other age groups. These are the major reasons that the young and middle-aged men tend to have the highest prevalence of ankle fractures. For women between 50 and 70 years of age, decrease of sex hormone after menopause may result in osteoporosis and decreased bone density and subsequently more and easy bone fractures. Appropriate measures are needed for elderly women to improve their osteoporosis and bone density to prevent possible bone fracture. Moreover, it is suggested that women after retirement in China get more active than younger women, and they consequently have engaged in more activities like dancing and exercise after retirement and may have more injury. Thirdly, decrease in the muscle strength and balance ability in old women may cause them to fall easily and consequently more fractures. In a study investigating population-based epidemiology of 9767 ankle fractures in north Denmark region^17^, it was found that the incidence of ankle fractures peaked among adolescents in both genders with a male predominance and that after the age of 19 years, the male incidence declined with age, but the female incidence of ankle fractures increased until 49 years. After the age of 50 years, the female incidence of ankle fracture continued to decrease. The differences in the incidence of ankle fractures between our study and theirs may be related to the ethnicity, traditions, local circumstances and weather. Women after retirement at age of 50 years in China are usually more active in daily activities engaging in dancing, household chores, looking after their grandchildren and travelling, and these low-energy activities may frequently cause low-energy ankle fractures. This was in consistent with findings in another study which investigated the epidemiology of adult ankle fractures during 2009–2013 in Sweden and demonstrated that over 2/3 of ankle fractures were caused by low-energy injury with a higher incidence in women who have a greater incidence primarily in the 30–60 year age range^18^. In men, the incidence of ankle fractures was more evenly distributed throughout their life^18^. Posterior malleolar fractures, fibular fractures at a higher location and Tillaux fractures can easily be missed in the diagnosis, and in this study, the missed diagnosis rate of these fractures was 1%. In posterior malleolar and Tillaux fractures, bone displacement was not apparent and could not be displayed. Moreover, these locations were usually ignored, consequently leading to missed diagnosis. If we recognized the possibility of fibular fractures at a higher location, additional radiography performed over the middle and upper segments of the fibula would be able to avoid missed diagnosis.

### Role of the Danis-Weber and the Lauge-Hansen classification systems

The Lauge-Hansen classification system has been traditionally applied for explaining the mechanism of injury based on x-ray morphology of the fracture^2,19^, however, several biomechanical studies conducted under more standardized conditions have questioned the mechanistic explanation of the fracture morphology provided by the Lauge-Hansen system^16,20,21^. Some recent studies have also demonstrated that the Lauge-Hansen classification system performed poorly for predicting the actual fracture mechanism^22,23^. One study investigating the x-ray features to predict ankle fracture mechanism found that the Lauge-Hansen classification system could predict supination injuries even though the study was on a small number of patients^2^.

Because of the limitations of the Lauge-Hansen classification system in mechanistic explanation of ankle fractures, the Danis-Weber and the Lauge-Hansen classification systems can be combined to analyze the mechanism of injury, location of fracture and facture line. The Danis-Weber classification system is mainly used for observing stability of the inferior tibiofibular ligament, and addition of the Lauge-Hansen system for staging can help doctors predict location and scope of injury, increase diagnosis accuracy and decrease the missed diagnosis rate. Danis-Weber type B and C fractures were mostly caused by external rotational forces, and at the time of injury, the foot was fixed while the body rotated resulting in a strong force with multiple ankle injuries and luxation. The use of the Lauge-Hansen classification system will help doctors predict fracture scope so as to avoid missed diagnosis. In Danis-Weber type B fracture, if the medial malleolus was found to be fractured, an injury might also occurr to the posterior malleolus where the posterior tibiofibular ligament is attached. In this case, if a plain radiograph did not show the fracture at the posterior malleolus, a CT scan should be suggested for precise display of the fracture at the posterior malleolus. If Danis-Weber type C fracture was suspected with or without accompanied increase of the inferior tibiofibular space while a fibular fracture was not found in the ankle joint, additional plain radiography should be performed over the middle and upper segments of the fibula so as to get rid of high-location fibular fracture. Another factor to miss a fibular fracture at a relatively high location in diagnosis is heavy symptoms of the initial injury so that symptoms at other locations are ignored. The Tillaux fracture can be easily missed and may need CT scanning for evaluation and confirmation because this type of fracture is usually hidden under images of adjacent bones in plain radiograph and has to be shown with three-dimensional imaging like CT scanning.

The key to avoid misdiagnosis is to combine the Danis-Weber and Lauge-Hansen classification systems in analyzing ankle fractures. The Danis-Weber classification system is based largely on the level of the fibular fracture and the associated level of lower tibiofibular ligament injury while the Lauge-Hansen classification system is mostly based on the position of the foot at the time of injury and the nature of the injurious force^11^. The Danis-Weber classification system is simple and can be used to concisely identify the degree of syndesmotic injury, whereas the latter system is generally applauded as being comprehensive in classifying the vast majority of ankle fractures besides being helpful for identifying associated ligament injuries and determining the severity of injury and degree of instability. With the Danis-Weber classification system, fine, non-apparent and occult fractures may be missed. The combination of these two systems may help avoiding misdiagnosis of ankle fracture. However, the disadvantage of the Lauge-Hansen system is its complexity and difficulty in clinical application and generalization. In our experience, the key point in clinical application of this system is to focus on the lower tibiofibular ligament injury. In type A Weber classification, the fibular fracture is located distally without injury of the lower tibiofibular ligament. In types B and C fractures, because the injurious force is mostly great and causes stage 4 fracture, the posterior malleolus (injury to the attachment of the posterior tibiofibular ligament) and anterior border of tibia (injury to the attachment of the anterior tibiofibular ligament) should be the key for evaluation and CT scanning is necessary for avoiding misdiagnosis.

### Fractures needing particular attention

Particular attention has to be paid to the following injuries of simple posterior malleolus fracture and simple medial malleolus fracture. For simple posterior malleolus fracture which exists only in the pronation-external rotation stage 4, injury to the lower tibiofibular ligament is certain with concomitant fibular fracture at a relatively high location, and additional radiographs at the middle and upper segments of the tibiofibula can demonstrate the fibular fracture. For simple medial malleolus fracture, three situations may exist. Firstly, simple medial malleolus fracture may be seen in supination-adduction stage 2, in which there is only simple injury to the lateral collateral ligament without lateral malleolus fracture or with only a small fracture line. In this type of fracture infrequently seen clinically, the location of medial malleolus fracture is relatively high caused by squeezing of the huckle-bone, no injury is present to the lower tibiofibular ligament, and the ankle joint is stable. Secondly, simple medial malleolus fracture may be seen in pronation-abduction stages 1–2 or pronation-external rotation stages 1–2, with these stages being difficult to distinguish. This fracture is stable rarely seen clinically. Thirdly, simple medial malleolus fracture may be seen in the pronation-external rotation stages 3–4, and this type of fracture is certainly concomitant with a fibular fracture at a relatively high location which can be demonstrated with additional radiographs at the tibiofibular middle and upper segments. In this type of fracture, particular attention should be paid to presence of posterior malleolus fracture, and small posterior malleolus fracture may be missed especially at the distal end.

### Limitations

There are some limitations in the present study. Not all patients had CT scanning. CT scanning was performed in patients with suspect of ankle fractures, in patients with no definitive diagnosis of fractures, or for planning of treatment in patients with definitive ankle fractures. In our study, a total number of 1085 patients had CT scanning, accounting for 27.5%. Not all patients had plain radiographs of the whole length of tibiofibula, and some small avulsion fractures and fibular fractures at a relatively high location may be missed. Not all ligament injuries were confirmed by magnetic resonance imaging or surgical operation. Consequently, these data were not analyzed in this study. With availability of CT scans for all patients, information on the fracture morphology would have become more detailed. Magnetic resonance imaging could provide more reliable information for ligamentous injuries. The single ethnicity of Chinese population is another limitation besides the single center study and retrospective design.

## Conclusion

Danis-Weber type B is the most common ankle fracture followed by Danis-Weber type A, type C and perpendicular compression fracture, with a similar incidence on the left and right side. Young and middle-aged men between 21 and 50 years and older women between 50 and 70 years have a greater incidence of ankle fractures, and combined application of the Lauge-Hansen and Danis-Weber classification systems can better help assessing the varied and complex ankle fractures and help doctors in predicting the injuries, increasing diagnostic precision and decreasing missed diagnosis.
